# On-Site Application of Solar-Activated Membrane (Cr–Mn-Doped TiO_2_@Graphene Oxide) for the Rapid Degradation of Toxic Textile Effluents

**DOI:** 10.3390/membranes12121178

**Published:** 2022-11-23

**Authors:** Maryam Yousaf, Mariam Akram, Ijaz Ahmad Bhatti, Muhammad Ahmad, Muhammad Usman, Muhammad Usman Khan, Abid Sarwar, Muhammad Sultan, Ihsanullah Sohoo

**Affiliations:** 1 School of Chemistry and Chemical Engineering, Beijing Institute of Technology, Beijing 102488, China; 2 Department of Chemistry, University of Agriculture Faisalabad, Faisalabad 38040, Pakistan; 3 Institute for Water Resources and Water Supply, School of Civil Engineering, Hamburg University of Technology, Am Schwarzenberg-Campus 1, 21073 Hamburg, Germany; 4 Department of Energy Systems Engineering, Faculty of Agricultural Engineering and Technology, University of Agriculture Faisalabad, Faisalabad 38000, Pakistan; 5 Department of Irrigation & Drainage, University of Agriculture Faisalabad, Faisalabad 38000, Pakistan; 6 Department of Agricultural Engineering, Bahauddin Zakariya University, Multan 60800, Pakistan; 7 Department of Energy and Environment Engineering, Dawood University of Engineering and Technology, New M.A. Jinnah Road, Karachi 74800, Pakistan

**Keywords:** photocatalysis, graphene oxide, dye degradation, solar-activated membrane, TiO_2_ nanowire

## Abstract

Solar-activated water treatment has become an emerging research field due to its eco-friendly nature and the economic feasibility of green photocatalysis. Herein, we synthesized promising, cost-effective, and ultralong-semiconductor TiO_2_ nanowires (NW), with the aim to degrade toxic azo dyes. The band gap of TiO_2_ NW was tuned through transition metals, i.e., chromium (Cr) and manganese (Mn), and narrowed by conjugation with high surface area graphene oxide (GO) sheets. Cr–Mn-doped TiO_2_ NWs were chemically grafted onto GO nanosheets and polymerized with sodium alginate to form a mesh network with an excellent band gap (2.6 eV), making it most suitable to act as a solar photocatalytic membrane. Cr–Mn-doped TiO_2_ NW @GO aerogels possess high purity and crystallinity confirmed by Energy Dispersive X-ray spectroscopy and X-ray diffraction pattern. A Cr–Mn-doped TiO_2_ NW @GO aerogels membrane was tested for the photodegradation of Acid Black 1 (AB 1) dye. The synthesized photocatalytic membrane in the solar photocatalytic reactor at conditions optimized by response surface methodology (statistical model) and upon exposure to solar radiation (within 180 min) degraded 100% (1.44 kg/m^3^/day) AB 1dye into simpler hydrocarbons, confirmed by the disappearance of dye color and Fourier transform infrared spectroscopy. An 80% reduction in water quality parameters defines Cr–Mn-doped TiO_2_ NW @GO aerogels as a potential photocatalytic membrane to degrade highly toxic pollutants.

## 1. Introduction

Water contamination is a major problem faced by the entire world, especially in developing countries. According to a rough estimation, around four billion people throughout the world have no or very little supply of clean water, and yearly, millions of people died due to drinking contaminated water [1,2,3]. Many industries, such as paper, pulp, dyestuff, pharmaceutical textiles, etc., are running throughout the world [4,5,6]. A huge number of pollutants are being discharged from these industrial processes that cause noticeable effects on the environment [7]. Around 20% of colored waste effluents are being discharged into water bodies from textile industries without treatment, which is badly polluting the environment. These colored effluents are rich in organic dye pollutants, and because of the non-biodegradable nature of these contaminants, they are a serious environmental risk and continuously disturb human life [8,9]. These organic dyes are not only aesthetically displeasing but also disturb the visual quality of water, which hinders light penetration. Moreover, dyes present in water reduce dissolved oxygen, ultimately affecting aquatic life. Humans, upon consuming this water contaminated with dyes, suffer from several diseases such as cancer, heart problems, respiratory diseases, diarrhea, jaundice (hepatitis), etc. Therefore, removing these dyes from the water before consumption is imperative to avoid health effects [10,11,12].

However, organic contaminants present in the industrial effluent are difficult to be removed by the natural degradation process owing to their biological and chemical stability [13,14]. So far, methods reported for the removal of organic dyes are ion exchange, adsorption, photocatalysis, chemical oxidation, coagulation, biodegradation, Fenton oxidation degradation, chemical reduction, and electrochemical treatment, etc. [15,16,17]. Unfortunately, the main drawbacks of these techniques are their low dye removal efficiencies, slow reaction rates, narrow operating pH range, the problem of the disposal of the spent contaminated activated sludge, and the control of the appropriate dye removal conditions [18,19,20]. Among these techniques, the removal of dyes from contaminated water using the advanced oxidation process (AOP), mainly photocatalysis, is considered to be the most efficient process. In recent years, substantial attention has been paid to the AOPs, which are characterized by the generation of a hydroxyl radical (^•^OH) by a nano-photocatalyst; nano-photocatalysts possess extraordinary properties due to the quantum confinement effect of nanomaterials [21,22,23]. The ^•^OH produced during photocatalysis possesses a high oxidation potential (estimated to be +2.8 V) relative to other oxidants, which is why it can oxidize the organic dyes [24,25].

Mechanistically, photocatalysis is a photo-induced process, during which, electron and hole pairs are generated by photocatalysts upon exposure to light, which in turn generates highly reactive ^•^OH radicals [26]. The photocatalytic efficiency of the photocatalyst can be enhanced by doping with metal and non-metal ions [27,28,29]. Different semiconductors oxides, such as TiO_2_, ZnO, ZnS, and WO_3_ have been used as photocatalysts [30,31]. Among the reported photocatalysts, TiO_2_, due to its strong oxidizing power, photostability high surface-to-volume ratio, and low toxicity, is considered a suitable candidate to be used as a photocatalyst. However, its wide band gap of 3.0–3.2 eV, UV light absorption capacity, and transparency to visible light restrict its broader application [32]. The crystalline structure of TiO_2_ has been modified by doping with metals (Fe, Zn, Cu, Ni, Cd, V) and non-metals (N, C) [33], lanthanides (Eu, Gd, La, Yb) [34], and noble metals (Ag, Pt, Au) to reduce its band gap energy suitable for visible light absorption [35,36,37]. The TiO_2_ band gap is modified by the crystalline modification by replacing the Ti atoms in the TiO_2_ crystal lattice by doping with different metals and non-metals with different charge/oxidation states, and the stability of the newly formed doped TiO_2_ is dependent upon the crystallite size [34]. Crystalline TiO_2_ can be prepared by the hydrothermal method, the extraction–pyrolytic method, and the precipitation method with variable properties [38,39].

However, compared to high-cost noble metals, the low cost and superior properties of transition metals show promise for doping and altering the electronic properties of TiO_2_ [40]. Another problem faced in photocatalysis is the rapid electron–hole pair recombination; this problem in TiO_2_ after band gap tuning can be overcome by conjugating with graphene oxide (GO). GO/TiO_2_ acts as a photocatalyst by efficiently degrading dye like Acid Black under UV and visible irradiation. The large surface area and oxygen-containing functional groups of GO facilitate the adsorption of dye on photocatalysts with high quantum efficiency to resist the recombination of the electron pair [41,42]. This conjugated mixture can be turned into a polymeric photocatalytic membrane by means of combining it with sodium alginate polymer in the form of aerogels. Aerogel has very unique properties such as its low density, high surface area, and porosity [43,44].

Keeping these facts in consideration, herein, the band gap of TiO_2_ was tuned by doping with the transition metals, i.e., chromium (Cr) and manganese (Mn). Cr and Mn exist in variable oxidation/charge states that can create an electron transport chain, facilitating the more feasible redox photocatalytic degradation process of dyes [37]. After co-doping with Cr–Mn, TiO_2_ was conjugated with GO to form a Cr–Mn-doped TiO_2_@GO composite that was polymerized into aerogel photocatalytic membrane for the solar-assisted photocatalytic degradation of Acid Black 1 dye. All operational parameters, i.e., oxidant concentration (H_2_O_2_), pH and sunlight exposure time, and the size of the aerogel to study the photo-degradation of Acid Black 1, were optimized by response surface methodology (RSM). Because of our synthesized Cr–Mn-doped TiO_2_@GO aerogel photocatalytic membrane’s large internal surface area, porosity, high optical properties, low density, and high adsorptive site due to GO functionalities, it favors the greater interaction of pollutants with the membrane leading to 100% degradation within 180 min of exposure to solar light. The employment of our designed photocatalytic membrane will be cost-effective because of the simultaneous adsorption and photodegradation of toxic organic pollutants [45,46,47].

## 2. Materials and Methods

### 2.1. Materials and Chemicals

Titanium oxide Anatase (TiO_2_), chromium nitrate (Cr(NO_3_)_3_), manganese nitrate (Mn(NO_3_)_2_), sodium chloride (NaCl), sodium hydrogen phosphate (NaHPO_4_), calcium chloride (CaCl_2_), sodium alginate (Na alginate), citric acid, hydrogen peroxide (H_2_O_2_), sodium hydroxide (NaOH), and hydrochloric acid (HCl) were used, which were obtained from the Radiation Lab. All reagents used were of analytical grade and used further without any purification.

### 2.2. Synthesis of Graphene Oxide (GO) and TiO_2_ Nanowire (NW)

Graphene oxide (GO) was synthesized by a bottom-up approach using the method reported in the literature with slight modification [48]. In brief, 2 g of citric acid (CA) was heated at 200 °C in heating in the mantle. Within 5 min of heating, the CA was liquified and changed color from white to pale yellow and finally turned to dark brown after three hours, indicating the formation of GO sheets. Later, the formed dark brown was removed from the mantel, cooled, and neutralized to pH 7 with 50 mL NaOH (0.1 M) solution. The resulting dark brown GO solution was stored for further use.

Cr–Mn-doped TiO_2_ nanowires (NW) were prepared according to the previously reported literature [49]. In brief, the molten salt flux method was used for the synthesis of the Cr–Mn-doped TiO_2_ NW. A total of 0.50 g of TiO_2_ powder was mixed with the 2.0 g of NaCl and 0.50 g of NaHPO_4_ in a 1:4:1 ratio, and 2.5% atomic percentage of Mn(NO_3_)_2_ and Cr(NO_3_)_3_ dopants. The whole reaction mixture was finely ground in a pestle mortar to form a fine powder mixture and then calcined in a crucible at the temperature of 825 °C for eight hours in a muffle furnace (ramping rate of 2 °C/min). After calcination, the prepared mixture was allowed to naturally cool down at room temperature, and then it was thoroughly washed with water to remove unreacted salts and dried in an oven at 80 °C for eight hours.

### 2.3. Synthesis of Cr–Mn-Doped TiO_2_/Graphene Oxide Aerogels

Individually synthesized GO and Cr–Mn-doped TiO_2_ NWs were used to prepare Cr–Mn-doped TiO_2_ NW @GO aerogels using the polymerization method. In brief, 2.5 mL (0.2 g/100 mL) of GO was taken and diluted with 48.5 mL of distilled water followed by the addition of 0.5 g/50 mL of Na alginate (at 50 °C) and 0.16 g of Cr–Mn-doped TiO_2_ NW; the whole reaction mixture was stirred for 20 min at room temperature to ensure polymerization. Later on, the gel was dropped into CaCl_2_ solution (1.0 M) followed by freeze-drying for 4–5 h to obtain Cr–Mn-doped TiO_2_ NW @GO aerogels. 

### 2.4. Characterization of Cr–Mn-Doped TiO_2_/Graphene Oxide Aerogels

The synthesized Cr–Mn-doped TiO_2_ NW @GO aerogel was characterized through X-ray diffraction (XRD, Jeol JDX-3532 diffractometer, Tokyo, Japan) to study the crystal structure of the aerogel. Scanning Electron Microscopy (SEM, Quanta 250, FEG (Waltham, MA, USA) analysis was conducted to determine the surface morphology. Energy Dispersive X-ray (EDX) analysis was performed to identify the elements present in the synthesized material. Fourier transform infrared spectroscopy (FTIR, Bruker IFS 125HR Japan, Tokyo, Japan) was conducted to identify the functional groups. The Brunauer–Emmett–Teller (BET) surface area and pore size analysis were performed by measuring the N_2_ adsorption–desorption isotherms.

### 2.5. Statistical Analysis

The optimization of the operational reaction parameters, i.e., the size of the aerogels, irradiation time, pH, and oxidant concentration, was performed using Design Expert 7 pro software under Response Surface Methodology (RSM). RSM includes different designs, these designs are three-level factorial, central composite (CCD), Box–Behnken (BBD), and D-optimal [50]. Here, we used RSM in combination with central composite design (CCD) to optimize four operational reaction parameters; these parameters were chosen as independent variables while the degradation rate of the dye was chosen as the output response variable. The list of variables, and their actual lower and higher levels for optimization are given in Table 1. CCD design (CCD) was adopted to evaluate the combined effect of the four independent variables by 30 sets of experiments. A general expression of the mathematical relationship describing the response of four independent variables (A, B, C, D) can be approximated by quadratic polynomial Equation (1):

Y = β + β_1_X_1_ + β_2_X_2_ + β_3_ X_3_ + β_4_X_4_ + β_12_X_12_ + β_2_X_2_+ β_33_X_32_ + β_44_X_42_ + β_12_X_1_X_2_ + β_13_X_1_X_3_ + β_14_X_1_X_4_ + β_23_X_2_X_3_ + β_24_X_2_X_4_ + β_34_X_3_X_4_ (1)

where Y presented the predicted response and β is the coefficient constant. While β_1_, β_2_, β_3_, and β_4_ are the linear effect coefficients, β_11_, β_22_, β_33_, and β_44_ are the quadratic effect coefficients, and β_12_, β_13_, β_14_, β_23_, β_24_, and β_34_ are the interaction effect coefficients. X1, X_2_, X_3_, and X_4_ are the independent variables corresponding to A, B, C, and D, respectively.

The data were analyzed by analysis of variance (ANOVA), and the mean values were considered of significant difference when *p* < 0.0001. The optimal values of the operational parameters were estimated by the three-dimensional response surface analysis of the independent variables and the dependent variable. 

### 2.6. Photocatalytic Degradation Potential of Acid Black Dye by Cr–Mn-Doped TiO_2_ NW @GO Aerogels

**Scheme 1:**
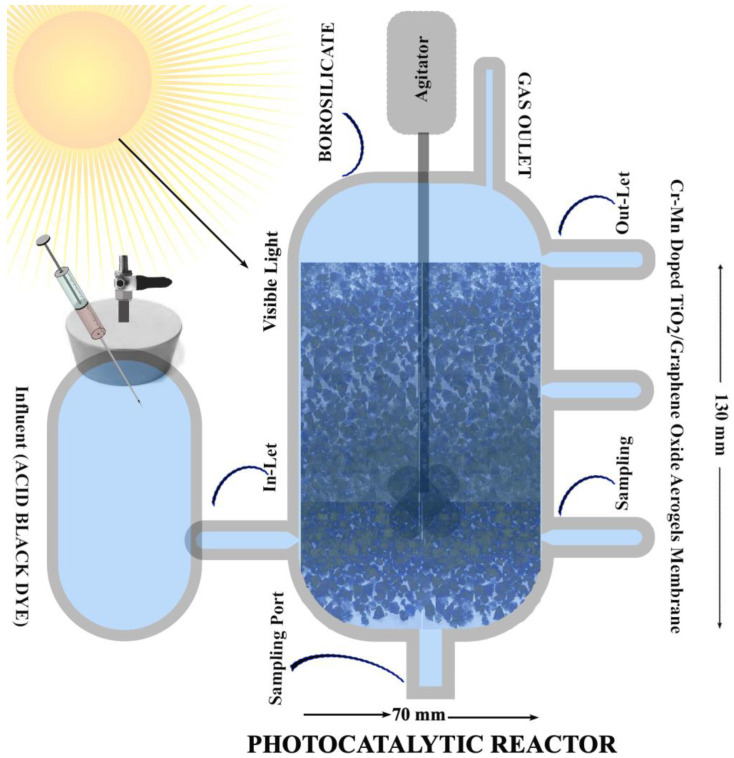
Schematics of the photocatalytic reactor design and experimental setup for the degradation of AB 1 dye.

Solar light-assisted photocatalytic degradation of Acid Black 1 dye (AB 1) (C_36_H_23_N_5_Na_2_O_6_S_2_) by Cr–Mn-doped TiO_2_ NW @GO aerogels photocatalyst was evaluated in borosilicate glass reactors (500 mL) filled with the Cr–Mn-doped TiO_2_ NW @GO aerogel membrane (12.5 mm thickness). A reaction mixture containing a 500 ppm solution of AB 1 dye was exposed to solar radiation (one Sun illumination, wavelength = 530 nm) at conditions optimized by RSM in the solar photocatalytic reactor (Scheme 1). The rate of the degradation was measured with a UV–Vis spectrophotometer (Maximum wavelength = 620 nm) followed by the degradation % calculation using Equation (2):

(Degradation (%) = (A_0_ − A_t_)/(A_0_) ∗ 100 (2)

where A**_o_**: Absorbance of Dye at Zero minutes, A_t_: Absorbance of Dye at time t.

The water quality parameters of the treated and untreated AB 1 dye-containing textile industrial effluents such as BOD, COD, and TOC were analyzed by using previously reported literature (Ahmad et al., 2019).

## 3. Results and Discussion

### 3.1. Characterization of Cr–Mn-Doped TiO_2_ NW @GO Aerogel 

**Figure 1:**
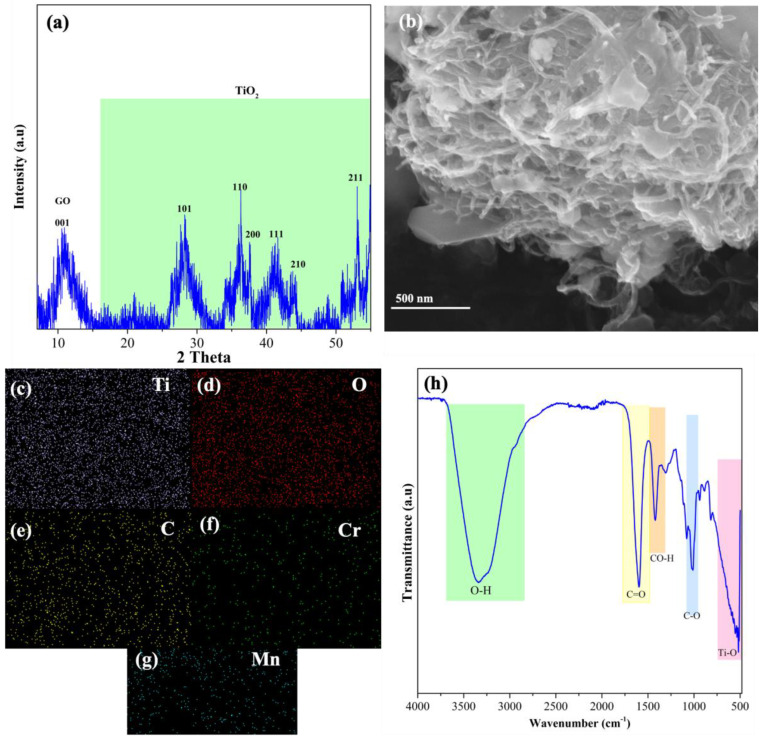
XRD crystallographic pattern (**a**), SEM micrograph (**b**), functional group analysis (**c**–**g**), and elemental analysis (**h**) of Cr–Mn-doped TiO_2_@GO aerogel.

The crystal structure of the prepared Cr–Mn-doped TiO_2_@GO aerogel was studied through XRD analysis (Figure 1a). The appearance of the XRD diffraction peaks at 2θ° values of 27.5°, (110), 36.04 (101), 41.31 (111), 54.28 (211), and 55.3° (102) confirmed the presence of the rutile phase of TiO_2_ [51], while the absence of diffraction peaks corresponding to the oxides or metallic phase of Cr–Mn dopants confirmed the successful substitution of Cr–Mn with the Ti in the TiO_2_ crystal structure. Furthermore, the poor crystalline nature of the dopant will favor enhanced oxygen vacancies and the catalytic nature of the Cr–Mn-doped TiO_2_@GO aerogel. Moreover, the appearance of a diffraction peak at 2θ = 10.9° corresponded to the (100) plane of GO [52], confirming the presence of GO and the successful formation of the Cr–Mn-doped TiO_2_ NW @GO aerogel.

In order to analyze the structure and morphology of synthesized Cr–Mn-doped TiO_2_@GO aerogel the SEM analysis was carried out (Figure 1b). A fine network of Cr–Mn-doped TiO_2_ NWs surrounding the nanosheets of GO can be clearly seen in SEM images, which confirmed that Cr–Mn-doped TiO_2_ NW is successfully grafted over GO nanosheets. During the polymerization process for the formation of aerogel, Cr–Mn-doped TiO_2_ NWs completely attached to the surface of GO sheets which makes it difficult to differentiate between the Cr–Mn-doped TiO_2_ NWs and the GO sheets. Further, the elemental analysis and purity of the Cr–Mn-doped TiO_2_@GO aerogel were confirmed by EDX (Figure 1c–g). It is quite obvious from Figure 1c–f that the Ti, O, and C (a component of GO) in the prepared sample are present in higher concentrations, while Mn and Cr, being the dopants, are present in a lesser concentration in the EDX mapping. All these elements are the main components of the prepared Cr–Mn-doped TiO_2_@GO aerogel.

The specific surface area (S_BET_) and porosity of the prepared Cr–Mn-doped TiO_2_@GO aerogel were evaluated with the help of N_2_ adsorption–desorption isotherms through BET. From the results, it was obvious that the incorporation of Cr–Mn in TiO_2_ enhanced its S_BET_ (79.3 m^2^/g) compared to the pure TiO_2_ (46.2 m^2^/g). This enhancement is attributed to the control of the crystal grain size due to the incorporation of dopants. The surface area was further increased upon the addition of GO and the formation of an aerogel structure (280.2 m^2^/g). These results confirmed that the synthesized materials are highly porous in nature (Table 2). The high porosity of aerogel provides a microchannel for the efficient interaction of pollutants with the Cr–Mn-doped TiO_2_@GO aerogel photocatalytic membrane. Moreover, the conjugation of Cr–Mn-doped TiO_2_ NW with the GO nanosheets will not only enhance the surface area for pollutant interaction but also facilitate the photo-generated electron–hole separation and their transmission between the GO nanosheets and Cr–Mn-doped TiO_2_ NW.

FTIR analysis of Cr–Mn-doped TiO_2_@GO aerogel shows (Figure 1d) the prominent broad peak from 500–1000 cm^−1^ corresponding to the Metal–O/Ti–O bonds in the crystal lattice and with the C of GO (Ti–O–C). A broad absorption peak at 3340 cm^−1^ was due to the stretching vibration of the O–H (hydroxyl) group of water molecules, which indicated the existence of water molecules. Additionally, the presence and conjugation of GO in the Cr–Mn-doped TiO_2_@GO aerogel is confirmed from the characteristic stretching vibration peaks at 1030 cm^−1^ (epoxy C–O), 1425 cm^−1^ (C–OH) and 1570 cm^−1^ (ketone or carboxylate C=O) [53]. These hydroxyl and other oxygenated functional groups present at the corners and on the basal planes of GO could form bonds with the Ti–O and with functional groups of alginate to form a robust Cr–Mn-doped TiO_2_@GO aerogel matrix. 

### 3.2. Statistical Evaluation of Photo Catalytic Activity of Cr–Mn-Doped TiO_2_@GO Aerogel 

The RSM was utilized to optimize the conditions for the rapid photodegradation of AB 1 dye. Three-dimensional response surfaces were generated by using the Design Expert 7 pro software, which presented the visual relationship between different variables [54,55]. The RSM results of 30 runs were obtained on the basis of polynomial Equations (1) and (3) and are provided in Table 3.

(Y) = +85.00 + 4.82 A + 6.17 B + 0.60 C − 1.55 D − 4.76 A B + 4.59 A C + 1.62 A D − 4.03 B C − 1.01 B D + 3.84 C D − 11.53 A2 − 7.53 B^2^ − 7.28 C2 − 4.98 D2 (3)

where ‘Y’ is the degradation (%), and the actual values of the variables A, B, and C are shown in the equation. The validated model shows the interactive effects of variables on the degradation % (Y) of AB 1 dye under sunlight radiation. The interaction of parameters is demonstrated by the AB, AC, and BC processes. The parameters were related by coefficients and the signs (+, −) in the model. The coefficients indicate the specific weight of the parameters in the model, while the signs (+) and (−) affect the synergistic and antagonistic effects of variables on the response (Y). The coefficients indicated the weight of the variables in the model, which determines the important roles of the parameters in the AB 1 dye degradation.

**Figure 2:**
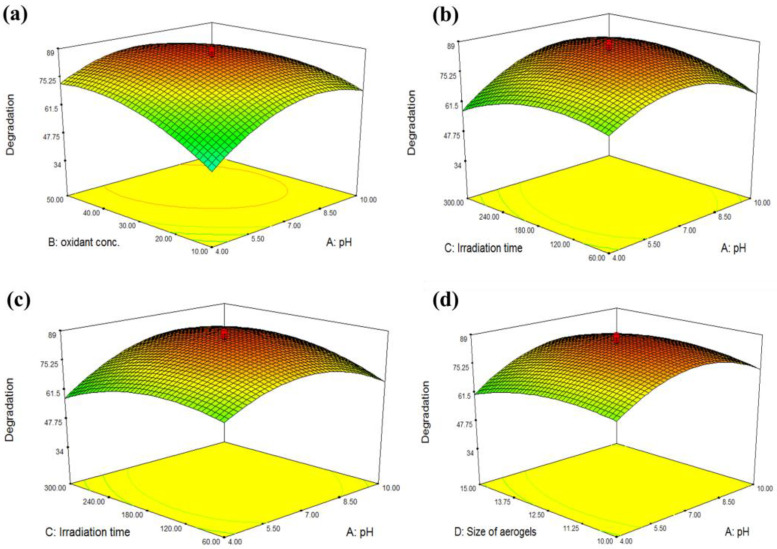
Interactive effects of different parameters in terms of 3D RSM plots, effect of pH and oxidant concentration (**a**), the effect of pH and irradiation time (**b**), the effect of oxidant concentration and irradiation time (**c**), and the effect of pH and the size of aerogels on the photocatalytic degradation of AB 1 dye (**d**).

According to the ANOVA, the *p*-value was less than 0.0001, which means that the model was significant; on the other hand, the *p*-value greater than 0.1000 showed that the model was not significant. Furthermore, the *p*-value of <0.0001 and the agreement of adj. R^2^ (0.9148) and R^2^ (0.9559) values with the reduced C.V. value of 8.34% showed that the model is highly significant, while the lack of a fit test showed good predictability. In the case of A, B, AB, AC, BC, and CD, A2, B2, C2, and D2 are significant model terms. The interactive effect of these variables in terms of response surfaces is shown in Figure 2. An ANOVA table was formed by applying design expert software for checking the AB 1 degradation rate from wastewater. If more values are insignificant, then the reduction in the model improves the model. The level of the f-value of the lack of fit applied to the model is not significant relative to the pure error. Hence, a non-significant lack of fit is good.

Three-dimensional surfaces graphically represent the interaction between two variables while the other variables are kept constant (Figure 2). pH and oxidant concentration are very sensitive factors that influence the degradation of AB 1; their interactive effect in terms of the 3D response surface over the AB 1 degradation is given in Figure 2a. As the value of the pH increases from acidic to neutral, the degradation of the dye is increased and reaches a maximum value at neutral pH. Upon further increasing the pH of the AB 1 dye sample, the degradation rate decreased. Therefore, pH 7 is optimum for the degradation of AB 1 dye. The rate of AB 1 degradation was enhanced from 1–3 mmol of oxidant concentration, and upon the further addition of oxidant concentration, the degradation rate decreased. 

The interactive effect of irradiation time and pH also influences the rate of AB 1 degradation (Figure 2b). An increase in irradiation time along with pH increased AB 1 degradation but up to a certain limit, i.e., neutral pH and an irradiation time of 180 min (Figure 2c), after which, more time did not affect the degradation rate. The size of the aerogel has a reasonable effect on the degradation rate of the dye (Figure 2d); upon an increment in the size of the aerogel, the % degradation of AB 1 dye is increased. However, upon a further increase in the aerogel size from 12.50 mm, no prominent effect in the rate of AB 1 degradation was observed. 

Hence, at the optimum aerogel size of 12.50 mm at neutral pH (7), maximum AB 1 degradation was achieved. Further increases in pH decreased the degradation rate to the extent to which the aerogel size was increased. Conclusively, according to the RSM response surfaces, the optimized parameters were pH = 7, solar irradiation time = 180 min, oxidant concentration = 1–3 mmol, and at an aerogel size of 12.50 mm, the maximum degradation of AB 1 dye was achieved. Higher or lower values than the optimized conditions led to decreases in the AB 1 degradation rate, either due to changes in the surface charge (pH effect), surface area change (size of aerogel), or the scavenging or self-decomposition of hydroxyl free radicals (effect of irradiation and oxidant) produced during photocatalysis that are responsible for AB 1 dye degradation.

### 3.3. Evaluation of Extent of AB 1 Dye Degradation by Cr–Mn-Doped TiO_2_@GO Aerogel 

**Figure 3:**
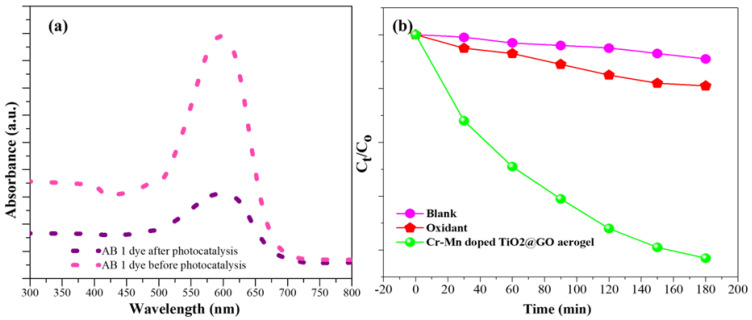
Evaluation of AB 1 dye degradation by UV/Vis spectrophotometer (**a**) before and after degradation using Cr–Mn-doped TiO_2_@GO aerogel photocatalytic membrane. Kinetics study of AB 1 dye degradation with and without Cr–Mn-doped TiO_2_@GO aerogel photocatalytic membrane (**b**).

The AB 1 degradation by a Cr–Mn-doped TiO_2_@GO aerogel photocatalytic membrane in the photocatalytic reactor was evaluated by comparing the UV/Vis spectra of AB 1 before and after treatment with a Cr–Mn-doped TiO_2_@GO aerogel photocatalytic membrane. The extent of the degradation of AB 1 was evaluated by UV/Vis spectroscopy at 620 nm, and the reduction in the peak corresponding to AB 1 dye after treatment with Cr–Mn-doped TiO_2_@GO aerogel photocatalytic membrane confirmed the efficient degradation of AB 1 dye (Figure 3a). Moreover, the AB 1 dye degradation was kinetically monitored (Figure 3b); it is quite obvious from the results that within 30 min of exposure to the Cr–Mn-doped TiO_2_@GO aerogel photocatalytic membrane, almost 50% of the dye was degraded, whereas in the case of blank (no membrane) and oxidant (no membrane), no remarkable AB 1 degradation was observed, confirming the efficient role of Cr–Mn-doped TiO_2_@GO aerogel photocatalytic membrane in AB 1 degradation, with complete removal at 180 min. 

**Figure 4:**
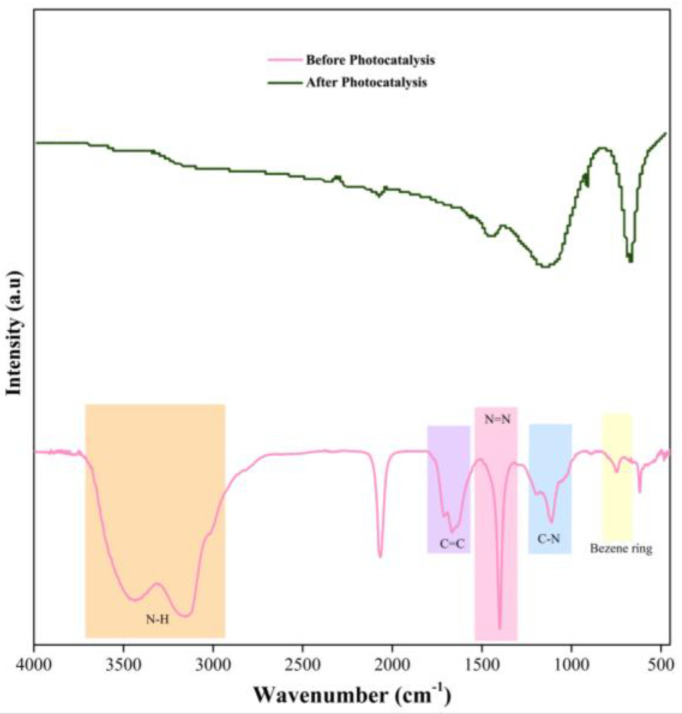
FTIR spectra of AB 1 dye wastewater before and after photocatalytic treatment with Cr–Mn-doped TiO_2_@GO aerogel photocatalytic membrane.

Further, FTIR of treated and untreated AB 1 dye solution was compared. From Figure 4, it is clear that peaks corresponding to AB 1 dye clearly vanished, confirming the efficient degradation of AB 1 dye in wastewater. Peaks corresponding to stretching vibrations associated with the N-H (3200–3400 cm^−1^), C-N (1000–1200 cm^−1^), and N=N azo bonds (1400–1490 cm^−1^) completely disappeared after treatment with the Cr–Mn-doped TiO_2_@GO aerogel photocatalytic membrane, confirming that dye is mineralized into hydrocarbons because of the absence of other aromatics, specifically aryl amines peak (1600–1650 cm^−1^) [56] as depicted in Figure 4.

**Figure 5:**
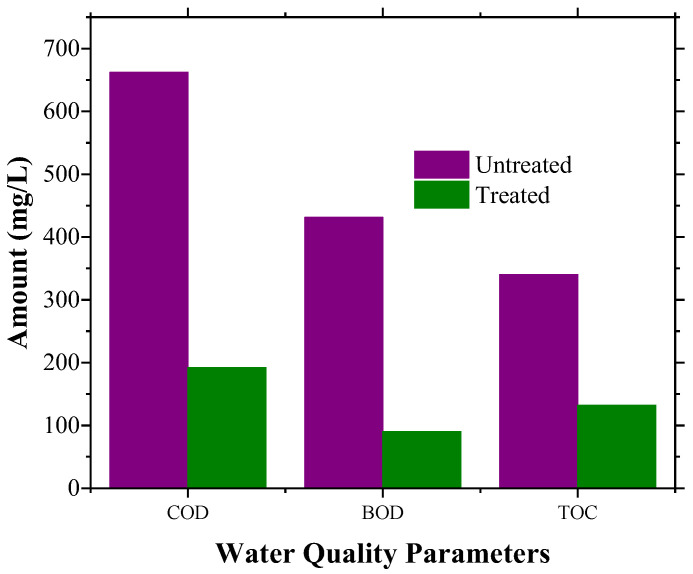
Water quality parameters evaluated before and after with Cr–Mn-doped TiO_2_@GO aerogel photocatalytic membrane.

A quantitative assessment of the degradation of the pollutants from water quality parameters has been analyzed before and after photocatalytic treatment with a Cr–Mn-doped TiO_2_@GO aerogel photocatalytic membrane. The % reduction in the COD, TOC, and BOD in wastewater has been measured. From Figure 5, it can be seen that COD is ~71%, BOD ~79%, and TOC ~61% reduced, confirming the high efficiency of the prepared Cr–Mn-doped TiO_2_@GO aerogel photocatalytic membrane.

### 3.4. Reusability of Cr–Mn-Doped TiO_2_@GO Aerogel Photocatalytic Membrane

**Figure 6:**
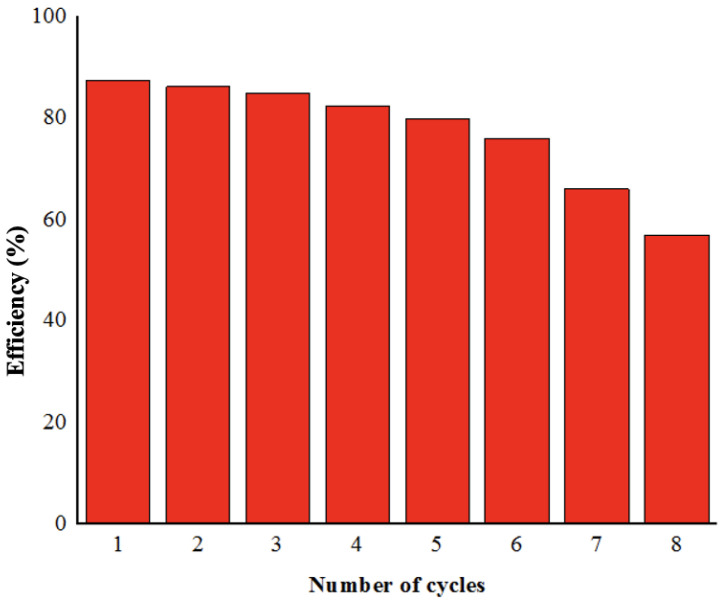
Reusability of the Cr–Mn-doped TiO_2_@GO aerogel photocatalytic membrane.

The Cr–Mn-doped TiO_2_@GO aerogel photocatalytic membrane can be facilely separated from the reaction system for recycling. The photocatalyst with the remarkable potential of AB 1 degradation can be easily separated from the solution and can be reused with unvaried performance. Herein, the results showed that as the number of cycles of reusability increased, initially, the efficiency of the catalyst was retained, and then it continuously decreased. However, even after eight cycles of reuse, the catalyst retained its efficiency of dye degradation of more than 50% in wastewater (Figure 6). Here, the first cycle means that catalyst has already been used. 

### 3.5. Mechanism of Degradation of AB 1 Dye by Cr–Mn-Doped TiO_2_@GO Aerogel

In the first step, (a) Cr–Mn-doped TiO_2_@GO aerogel photocatalytic membrane, upon exposure to solar light radiation, generates an electron–hole pair (e^−^h^+^). h^+^ is located in the valence band (VB) that is formed upon the excitation of e^−^ from the VB to the conduction band (CB); the availability of both e^−^ and h^+^ causes the redox reaction to take place [35,57]. In the second step (b), the Cr–Mn-doped TiO_2_@GO aerogel adsorbed the water molecules through GO functional groups. These water molecules become oxidized by the h^+^, resulting in the formation of hydroxyl ions (OH^−^). In the third step, (c) OH^−^ upon further oxidation formed a hydroxyl radical (^•^OH), which attacks AB 1 dye, leading to its degradation into lower hydrocarbons through an oxidation reaction. Another possibility (d) of dye degradation is attributed to the presence of e^−^ at the CB. This e^−^ will reduce the O_2_ molecule at the CB, resulting in the formation of ^•^OH and superoxide anion free radicals (O_2_^−^), leading to the reductive degradation products of AB 1 dye.

(a) Redox reaction involved in photocatalytic degradation of AB 1 dye by Cr–Mn-doped TiO_2_@GO aerogel photocatalytic membrane:

Cr–Mn-doped TiO_2_@GO aerogel + hυ → Cr–Mn-doped TiO_2_@GO aerogel (e^−^_CB_ + h^+^_VB_) (a)

h^+^_VB_ + H_2_O → H^+^ + OH^−^ (b)

h^+^_VB_ + OH^−^ → ^•^OH + AB 1 dye → Lower hydrocarbons +CO_2_ + H_2_O+ Gases (c)

(Oxidative removal of dye) →

e^−^_VB_ + O_2_/H_2_O → ^•−^O_2_ + H_2_O_2_ → ^•^OH → Reductive degradation of AB 1 dye (d)

(b) Overall reaction:

Cr–Mn-doped TiO_2_@GO aerogel + ^•^OH/^•−^O_2_ + AB 1 dye → Oxidation/reduction products + gases

Moreover, GO supports the Cr–Mn-doped TiO_2_ and prevents charge recombination by enhancing the charge transfer to the AB 1 dye, leading to more efficient, rapid, and effective AB 1 dye degradation. Because rapid charge recombination slows down or even stops the photocatalytic activity, the presence of GO facilitates the charge separation along with the charge transfer at the surface, thus enhancing the reaction kinetics and rate of degradation of the dye. Because photocatalysis is a surface phenomenon, increased surface area leads to enhanced photocatalysis. The incorporation of GO enhanced the surface area of Cr–Mn-doped TiO_2_. Further, ^•^OH radical generation and the availability of active sites are proportional to the enhanced surface area. All these factors helped the Cr–Mn-doped TiO_2_@GO aerogel photocatalytic membrane to degrade upon exposure to solar radiation.

## 4. Conclusions

TiO_2_ doping is among the hot topics, due to its high catalytic performance toward the mitigation of environmental pollution; however, the band gap reduction and separation of charge holes require strategic modification by the incorporation of multiple metals. TiO_2_ doping with Cr/Mn could reduce the band gap but has fewer adsorption capacities to selectively remove organic pollutants, especially when found in an aqueous form. GO has great potential to overcome this challenge due to its excellent absorption properties, and its conversion to an aerogel-based membrane could expose most of the reactive sites of all elements. The prepared Cr–Mn-doped TiO_2_@GO aerogel photocatalytic membrane possesses the ability to directly absorb solar radiation to degrade dyes. 

Herein, the Cr–Mn-doped TiO_2_@GO aerogel photocatalytic membrane was successfully synthesized by the polymerization method using sodium alginate as a linker. Characterizations of the Cr–Mn-doped TiO_2_@GO aerogel photocatalytic membrane proved it to be pure and highly crystalline, and it successfully grew over the GO nanosheets. The hydrophilic nature and higher photocatalytic properties of the Cr–Mn-doped TiO_2_@GO aerogel photocatalytic membrane successfully degraded AB 1 dye in textile industry wastewater upon exposure to solar radiation, as confirmed by FTIR analysis, a kinetics study of AB 1 dye degradation, and a UV–Visible spectrophotometer. Moreover, a clear ~80% reduction in COD (reduction), BOD, and TOC were observed that defines the Cr–Mn-doped TiO_2_@GO aerogel photocatalytic membrane as a potential material for the economical and efficient remediation of toxic pollutants released in textile industry effluents to make that water reusable.

## Figures and Tables

**Table 1:** CCD with predictive values and their experimental results.

Factor	Variables	Unit	Low Actual	High Actual
A	pH		4	10
B	Oxidant concentration	mmol	10	50
C	Irradiation Time	min	60	300
D	Size of aerogels	mm	10	15

**Table 2:** BET-specific surface area and pore size of Cr–Mn-doped TiO_2_ NW @GO aerogel.

Sr. No		TiO_2_ NW	Cr–Mn-Doped TiO_2_ NW	Cr–Mn-Doped TiO_2_ NW @GO Aerogel
1	S_BET_ (m^2^/g)	46.2	79.3	280.2
2	Pore volume (cm^3^/g)	0.135	0.216	0.35

**Table 3:** CCD with predictive values and their experimental results.

Run	A	B	C	D	Degradation (%)
1	7	70	180	12.50	70
2	4	10	300	15	42
3	13	30	180	12.50	50
4	7	30	180	7.50	76
5	7	30	180	12.50	89
6	4	50	60	15	59
7	10	10	300	10	58
8	7	7	60	12.50	55
9	4	10	60	15	32
10	10	10	60	10	51
11	10	10	300	10	62
12	10	10	60	15	43
13	10	50	300	15	56
14	10	50	60	15	58
15	7	30	160	10	87
16	10	30	180	10	58
17	7	30	60	12.50	82
18	7	30	180	7	78
19	7	30	300	12.50	63
20	7	10	420	15	46
21	4	50	180	10	50
22	4	50	60	15	70
23	7	30	300	12.50	87
24	7	30	300	12.50	86
25	10	10	60	10	89
26	4	50	80	10	49
27	1	30	300	10	34
28	4	10	60	15	30.2
29	7	30	60	12.50	60.4
30	4	10	180	15.23	41.7

## Data Availability

The data are contained within the article.
